# Consistent proportional macronutrient intake selected by adult domestic cats (*Felis catus*) despite variations in macronutrient and moisture content of foods offered

**DOI:** 10.1007/s00360-012-0727-y

**Published:** 2012-12-12

**Authors:** Adrian K. Hewson-Hughes, Victoria L. Hewson-Hughes, Alison Colyer, Andrew T. Miller, Simon R. Hall, David Raubenheimer, Stephen J. Simpson

**Affiliations:** 1 WALTHAM® Centre for Pet Nutrition, FreebyLane, Waltham-on-the-Wolds, Melton Mowbray, Leicestershire LE14 4RT UK; 2 Institute of Natural Sciences, Massey University, Albany, 0622 Auckland, New Zealand; 3 School of Biological Sciences and the Charles Perkins Centre, University of Sydney, Heydon-Laurence Building A08, Sydney, NSW 2006 Australia

**Keywords:** Macronutrient regulation, Nutritional geometry, Right-angled mixture triangles, Carnivore nutrition, Domestic cat

## Abstract

We investigated the ability of domestic cats to regulate the macronutrient composition of their diet when provided with foods that differed not only in macronutrient content but also in texture and moisture content, as typically found in the main forms of commercially manufactured cat foods. Cats were provided with foods in different combinations (1 wet + 3 dry; 1 dry + 3 wet; 3 wet + 3 dry) in three separate experiments. Within each experiment cats were offered the wet and dry food combinations in two (naïve and experienced) diet selection phases where all the foods were offered simultaneously, separated by a phase in which the foods were offered sequentially in 3-day cycles in pairs (1 wet with 1 dry). Using nutritional geometry we demonstrate convergence upon the same dietary macronutrient composition in the naïve and experienced self-selection phases of each experiment as well as over the course of the 3-day cycles in the pair-wise choice phase of each experiment. Furthermore, even though the dietary options were very different in each of these experiments the macronutrient composition of the diets achieved across all experiments were remarkably similar. These results indicate that a mammalian obligate carnivore, the domestic cat, is able to regulate food selection and intake to balance macronutrient intake despite differences in moisture content and textural properties of the foods provided.

## Introduction

In order to meet its nutrient requirements, an animal is faced with the seemingly simple task of eating food. But foods are not simply parcels of nutriment; they are complex mixtures of nutrients, water and other chemical components. Some of these components add bulk to the food and change its physical characteristics; others are ‘anti-nutritional’, being toxic or interfering with the palatability of the food or the availability of nutrients to digestion (Rosenthal and Berenbaum 1991; Provenza et al. 2003); and yet others can be medicinal (Huffman 2001, 2003; Villalba et al. 2006; Raubenheimer and Simpson 2009). In addition, animals in their natural environment may be faced with a number of food sources which differ in quality (i.e. nutritional and non-nutritional content) as well as quantity (availability) leaving the animal with the problem of deciding ‘what’ and ‘how much’ to eat.

It would appear that natural selection has been successful at solving this problem from the animals’ perspective with extensive evidence showing that animals across a variety of taxa including insects, fish and mammals regulate and balance their intake of nutrients by adjusting their choice of foods and the amounts eaten (Raubenheimer and Simpson 1993, 1997, 2003; Simpson and Raubenheimer 2000, 2012; Rubio et al. 2003; Sánchez-Vázquez et al. 1999; Felton et al. 2009). Even under artificial selection (domestication), where the diet of the animal today is largely determined by humans, the evidence is unequivocal that when provided with a choice of foods with different nutritional profiles both companion animals (e.g. cats and dogs) and livestock (e.g. pigs, poultry and mink) consume different amounts of the foods to balance their nutrient intake (Kyriazakis et al. 1991; Hewson-Hughes et al. 2011; Raubenheimer and Simpson 1997; Romsos and Ferguson 1983).

Domestic cats are often fed manufactured pet foods which are produced in two main formats, dry (i.e. kibbles/biscuits; ~7–10 % moisture) and wet (i.e. in cans or pouches; ~75–85 % moisture). We previously investigated the ability of cats to regulate macronutrient intake when provided with a choice of dry foods or wet foods and demonstrated that cats have a ‘target’ intake of approximately 52 % of total energy as protein, 36 % as fat and 12 % as carbohydrate (Hewson-Hughes et al. 2011). This target was only attainable by cats offered the wet foods since the macronutrient compositions of the dry foods did not span this region of nutrient space (the foods contained a minimum of 26 % energy from carbohydrate), although cats did mix diet compositions from the dry foods provided that approached as closely as possible the target selected by cats offered wet foods.

As well as differing in water content and texture, the typical macronutrient compositions of these formats are also quite different with dry foods typically having a higher carbohydrate content compared to wet foods as complex carbohydrates, mainly starches, are widely used as binding agents in the manufacture of dry feeds for domestic animals. Whereas herbivores are adapted to deal with complex carbohydrates in plants, there is evidence that excessive starch content in manufactured feeds for carnivores can compromise the attainment of a balanced complement of other nutrients—as reported, for example, in salmonid fish under aquaculture (Ruohonen et al. 2007) and suggested for domestic cats (Hewson-Hughes et al. 2011); our data indicated that cats have a limit to the amount of carbohydrate they will ingest (~300 kJ per day) which limited further food intake and which we termed the ‘carbohydrate ceiling’ (Hewson-Hughes et al. 2011). Accordingly, cats confined to high-carbohydrate foods (>50 % energy from carbohydrate) were left with a shortfall in protein and fat intake (relative to the target), potentially encouraging them to seek those nutrients elsewhere in the urban environment (Hewson-Hughes et al. 2011).

Although these results clearly demonstrated that cats were able to regulate their macronutrient intake when provided with a choice of foods of the same format (i.e. wet or dry) it is not known to what extent differences in physical properties (e.g. texture/hardness) and water content may influence food selection and macronutrient intake when these formats are offered together. Here, we describe a series of experiments in which cats were offered different combinations of wet and dry foods representing an overlapping series of nutritional compositions in order to investigate this.

## Materials and methods

### Animal housing and welfare

Adult, neutered domestic short hair cats (*Felis catus*) of both sexes bred and housed at the WALTHAM^®^ Centre for Pet Nutrition (WCPN), Melton Mowbray, Leicestershire, UK, participated in these diet selection experiments. Throughout each study the cats were housed and fed individually in purpose-built, behaviourally enriched lodges (*w* × *d* × *h*: 1.1 m × 2.5 m × 2.1 m) and were socialised as a group for approximately 1 h each day and had access to drinking water at all times. The cats were housed in social groups when not participating in experiments. The studies were approved by the WALTHAM^®^ Ethical Review Committee.

### Diets and general protocols

In this series of experiments cats were offered different combinations of wet and dry foods together with the aim being to determine the macronutrient balance selected by cats when offered foods not only with different macronutrient content but also different textures and moisture levels.

Four wet-format diets were manufactured using standard processing (canning) conditions at Mars Petcare, Saint Denis de l’Hôtel, France, based on Mars Inc. commercial recipes with the inclusion levels of chicken breast, soya protein isolate, lard and wheat flour altered to achieve differences in the macronutrient energy ratios of the diets (Table 1). Four dry-format diets were manufactured using standard processing (extrusion) conditions based on Mars Inc. commercial recipes with the inclusion levels of poultry meal, maize gluten, ground rice, wheat flour and beef tallow altered to achieve differences in the macronutrient energy ratios of the diets (Table 1). Both wet and dry diets were formulated to be complete and balanced according to the National Research Council and Association of American Feed Control Officials guidelines for adult feline maintenance.

**Table 1:** Nutrient compositions of wet and dry foods used in the experiments

Nutrient (g/100 g)	Wet foods					Dry foods				
	W	Wa	Wb	Wc	Wd	D	Da	Db	Dc	Dd
Moisture	82.1	80.6	80.9	80.8	83	7.7	7	7.9	7.2	5.2
Protein	10.3	7.7	13.3	9.6	10.2	39.4	24.8	49.4	26.9	41.5
Fat	4.2	2.9	3.3	6.7	3.9	12.4	9.5	9.5	27.3	18.8
Crude fibre	ND	ND	ND	ND	0.2	1	1.1	1.4	1.3	1.6
Ash	2.4	1.7	1.9	2.4	1.8	9.6	5.2	9.3	6.1	8.5
Carbohydrate	0.76	6.86	0.36	0.86	0.9	29.9	52.4	22.5	31.2	24.4
ME (MJ/kg)	3.16	3.08	3.31	3.86	3.03	14.55	14.68	13.9	18.21	16.33
PER (%)	53	41	66	40	55	40	25	52	22	37
FER (%)	43	30	32	56	41	30	23	24	53	41
CER (%)	4	29	2	4	4	30	52	24	25	22

The following modified Atwater factors were used to calculate the metabolisable energy (ME) content of the dry (protein, 14.64 kJ/g; fat, 35.56 kJ/g; carbohydrate 14.64 kJ/g) and wet (protein, 16.32 kJ/g; fat, 32.22 kJ/g; carbohydrate 12.55 kJ/g) foods

*ND* not determined, *PER* protein to energy ratio, *FER* fat to energy ratio, *CER* carbohydrate to energy ratio

Detailed experimental designs are given below, but in general, the cats received 150 g of each dry food from 10:30 to 08:30 h the following morning and for wet foods, 190 g of each diet was offered from 10:30 to 15:00 h and was replaced with a fresh aliquot (190 g) from 15:00 to 08:30 h the next day. Food intake for each cat was determined at the end of each feeding period (i.e. at 15:00 and 08:30 h for the wet foods and at 08:30 h for dry foods) as the difference between the mass of food offered (g) and the mass of food remaining (g). Each experiment consisted of three phases.

#### Phase 1: naïve simultaneous self-selection (NSS)

For 7 days, cats were exposed to all of the experimental foods simultaneously. The aim of this phase was to measure nutrient self-selection by the cats when naïve to the experimental foods. To avoid positional bias, the position of each food was rotated daily.

#### Phase 2: pair-wise self-selection

Cats were cycled through eight 3-day periods during which they were confined to a different wet and dry food-pair on each of the 3 days. The aim of this phase was to determine the nutrient balance selected within the various ‘restricted’ food-pair choices available to them each day. This phase also served as a conditioning phase in which the cats gained further experience of the foods.

#### Phase 3: experienced simultaneous self-selection (ESS)

In this phase, the regimen of phase 1 was repeated on the now ‘experienced’ cats.

#### Experiment 1: one wet and three dry foods

Eighteen neutered adult cats (9 male, 9 female), aged 2.0–9.1 years (mean ± SEM, 4.3 ± 0.4 years) and weighing 5.49 ± 0.24 kg were used in this experiment. The cats were offered one wet food (W, Table 1) with three dry foods (Da–c, Table 1) simultaneously in separate bowls during the NSS and ESS phases (phases 1 and 3). For the pair-wise selection (phase 2), the wet food was paired with each one of the dry foods over the course of each 3-day cycle (e.g. cycle 1, day 1, W + Da (pair A); day 2, W + Db (pair B); day 3, W + Dc (pair C); repeated 8 times in total).

#### Experiment 2: one dry and three wet foods

Seventeen neutered adult cats (9 male, 8 female; different cats to those used in experiment 1) aged 2.1 – 9.1 years (4.3 ± 0.4 years) and weighing 5.27 ± 0.26 kg were used in this experiment. The design of this experiment was the same as experiment 1 except here the cats were offered a single dry food (D, Table 1) together with three wet foods (Wa–c, Table 1) in the NSS and ESS phases and food D paired with each of the wet foods during each cycle of the pair-wise selection phase (D + Wa (pair A); D + Wb (pair B); D + Wc (pair C).

#### Experiment 3: three wet and three dry foods

Ten cats (4 male, 6 female), aged 2.9–9.8 years (5.27 ± 0.6 years) and weighing 4.61 ± 0.27 kg, that had previously taken part in experiment 1 (4 cats) or 2 (6 cats) were used in this experiment. For the NSS and ESS phases of this experiment cats were simultaneously offered 3 wet foods (Wa–c, Table 1) and 3 dry foods (Da–c, Table 1) in six separate bowls. The pair-wise choices offered during phase 2 were Wa + Da (pair A), Wb + Db (pair B) and Wc + Dc (pair C).

#### Experiment 4: one wet and one dry food

Having investigated the ability of cats to balance macronutrient intake when provided with combinations of wet and dry foods offered in differing ratios (i.e. relative number of bowls of each, 1 wet:3 dry; 3 dry:1 wet; 3 wet:3 dry) in experiments 1–3; here cats were offered a combination of foods more likely to be faced by cats in a domestic setting—one wet and one dry food. The foods were nutritionally complementary relative to the ‘intake target’ previously described in cats offered only wet foods (Hewson-Hughes et al. 2011) to determine whether providing different formats of food affected the ability of cats to achieve their target intake.

This experiment used commercially available wet (Sheba^®^ chunks in jelly, Turkey and Chicken variety; Wd, Table 2) and dry diets (Whiskas^®^ TOP, Chicken variety; Dd, Table 2). Twelve individually housed cats (6 males, 6 females aged 2.0–8.8 years (4.4 ± 0.6 years) and weighing 5.28 ± 0.26 kg) were provided with both foods simultaneously (in separate bowls) for 2 × 1 h periods each day for 12 days (75 g Dd + 190 g Wd at 09:00–10:00 h and 75 g Dd + 190 g Wd at 14:00–15:00 h). At the end of each feeding period, the mass of uneaten food was recorded and the amount eaten calculated as the difference between food offered and food remaining.

**Table 2:** Mean macronutrient intake (g/day) in the naïve and experienced self-selection phases across experiments 1–3

Experiment	Phase	Protein	Fat	Carbohydrate
1	NSS	44.5 (39.4–49.6)	17.4 (15.3–19.5)	16.5 (11.8–22.9)
	ESS	41.8 (36.6–47.0)	16.3 (14.1–18.5)	13.5 (9.6–18.8)
2	NSS	45.9 (40.7–51.1)	17.5 (15.7–19.3)	20.4 (15.7–26.4)
	ESS	45.0 (40.1–50.0)	16.7 (15.0–18.4)	19.5 (15.1–25.0)
3	NSS	40.2 (33.8–46.7)	18.5 (14.3–22.6)	19.4 (13.6–27.8)
	ESS	35.9 (29.7–42.1)	16.6 (12.6–20.6)	16.1 (11.1–23.3)

Mean macronutrient intakes are shown with 95 % confidence intervals in parentheses

### Statistical analyses

The outcomes analysed were total energy consumed and the % energy from each macronutrient as a proportion of total energy intake [i.e. protein: energy ratio (PER), fat: energy ratio (FER) and carbohydrate: energy ratio (CER)]. Mixed model analyses were used to analyse these outcomes to take into account the repeated measures on an individual cat when estimating the variance structure.

Experiments 1–3 were analysed individually and collectively; for individual experiments phase nested in cat were defined as random effects and phase defined as the fixed effect; for combined analysis of all three experiments, phase nested in cat nested in experiment were defined as random effects and experiment defined as the fixed effect. Data from the pair-wise selection phase were defined differently in the models for the PER/FER/CER and total energy intake analyses. There was no sense in determining whether the PER/FER/CER selected in each diet pairing was statistically different from each other as the compositions of the foods in each pairing would have made this the case. Instead, the average PER/FER/CER (Pair Average) over the three pair-wise choices was compared statistically to the average PER/FER/CER selected during the NSS and ESS phases. In contrast, it was of interest to know whether total energy intake was different depending on the diet pair offered and compared to the energy intake in the NSS or ESS phases and so for these analyses the average energy intakes for each pair-wise choice were compared separately to the values for NSS and ESS.

Experiment 4 was analysed by mixed models with cat as a random effect, to form summaries of overall means (i.e. no fixed effect was fitted).

Differences between levels within the fixed effects were tested at the overall 5 % level using Tukey honestly significant difference tests to adjust for multiple comparisons. All analyses were performed in R v2.13 statistical software (http://www.R-project.org/), with ‘lme4’ and ‘multcomp’ packages (R Development Core Team 2010).

In addition, simulation analyses were performed to determine whether the macronutrient profiles determined in experiments 1–3, pooled over all phases, were significantly different from profiles that would have resulted from random intake of the foods provided. Thus, food intakes (g) were first simulated assuming a total average food intake of 400 g (sd 100 g). The proportion of food eaten from each bowl was also simulated assuming an equal intake from each bowl on average (e.g. where four bowls were offered simultaneously the proportion of intake was simulated to be 25 % on average). From these simulations, the relative amount eaten (g) and the resulting PER, FER and CER of the diet composed were calculated for each meal in the design of each experiment. These simulations were performed 1,000 times and for each simulation the average PER, FER and CER were calculated using the previously described mixed model analyses. The proportion of simulated PER, FER or CER averages that were greater than or less than the experimental PER, FER or CER averages actually selected provided a significance test (GenStat^®^ v14 statistical software, GenStat VSN International, Hemel Hempstead, UK.).

## Results

### Experiment 1: one wet and three dry foods

**Fig. 1:**
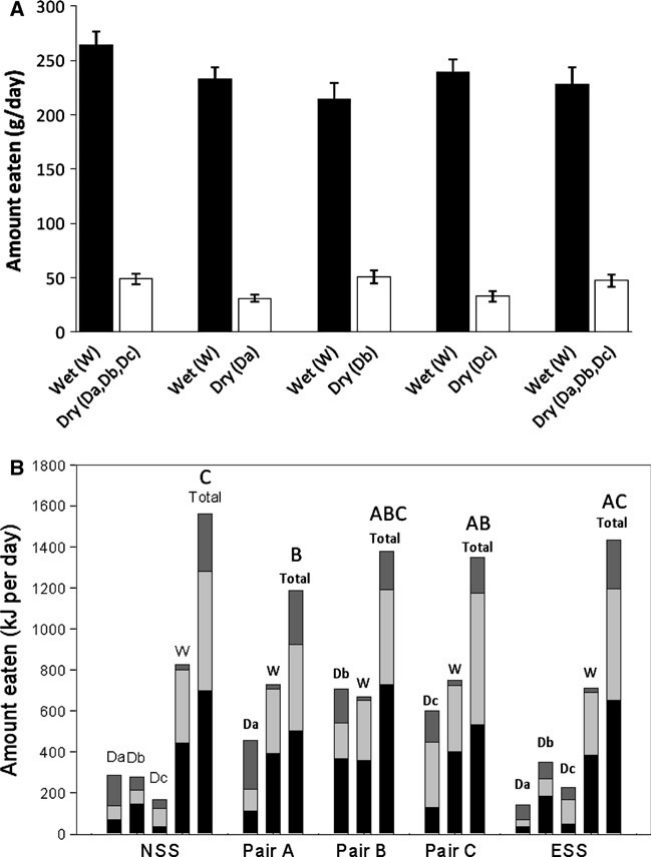
**a** Mean (±95 % confidence interval) food intake and **b** mean amounts of protein (*black*), fat (*light grey*) and carbohydrate (*dark grey*) energy ingested from the wet (W) and dry (Da, Db, Dc) foods offered to cats during the naïve self-selection (NSS), pair-wise choices (Pair A, Pair B, Pair C) and experienced self-selection (ESS) phases in experiment 1. Letters *A*–*C* *above the* *Total* bar (**b**) indicate statistically homogenous groups for total energy intake (i.e. energy intake in phases with the *same letter* are not significantly different)

**Fig. 2:**
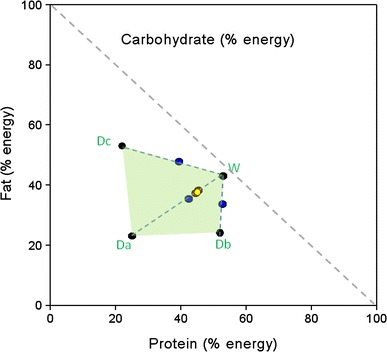
Right-angled mixture triangle (RMT) plot for experiment 1. In RMT plots the *X* and *Y* axes are read as normal; a third axis is shown as the hypotenuse (i.e. the *dashed grey line* representing carbohydrate in this figure). The values for this axis are read as 100 %—*Z* where *Z* is the value at which the diagonal with slope −45° through the point of interest intercepts the *X* and *Y* axes. For example, a −45° diagonal line through point Db would intersect the *X* and *Y* axes at 76 % and therefore give a carbohydrate value of 24 %. *Black circles* represent the proportional composition of protein, fat and carbohydrate in wet (W) and dry (Da–Dc) experimental foods. The *green shaded region* shows the area accessible to self-selecting cats with simultaneous access to the dry and wet foods. *Red circles* show the intake points selected in the naive and experienced self-selection phases and *blue circles* show the macronutrient profile of the diet composed during the pair-wise choices phase (each pair-wise choice is joined by a *dotted blue line*). The diet macronutrient composition averaged over each of the 3-day cycles of the pair-wise selection phase is indicated by the *yellow circle*

As can be seen in Fig. 1a the quantity (grams) of wet food consumed was greater than the quantity of dry food consumed by the cats in each phase of the experiment, comprising ~85 % of total food intake, regardless of whether there was one bowl of wet food and three bowls of dry food offered (as in the NSS and ESS phases) or one bowl of wet and one bowl of dry food offered (as in the pair-wise phase). This provides evidence that the cats did not just eat a similar quantity of food from each bowl (which might be expected if the cats selected food at random) and was supported by the simulation analyses which showed that the macronutrient profile of the diet composed by cats was significantly different to the profile that would have resulted from random food intake (*p* < 0.001). The patterns of food intake are depicted in terms of macronutrient energy intakes derived from each food and as total energy intake across all phases in Fig. 1b. Total macronutrient intakes in the NSS and ESS phases amounted to 697 kJ [95 % confidence interval (CI) 621 to 773 kJ] and 651 kJ (575–728 kJ) of protein, 582 kJ (508–656 kJ) and 547 kJ (471–624 kJ) of fat and 236 kJ (167–332 kJ) and 192 kJ (136–272 kJ) of carbohydrate, respectively. The macronutrient composition (expressed as % of total energy intake) of the self-selected diets composed by naïve and experienced cats (red dots) as well as the resulting diet composition of each of the pair-wise choices (blue dots) are plotted in a right-angled mixture triangle (RMT) (Raubenheimer 2011; Fig. 2). The macronutrient profile of the diets composed in the NSS and ESS phases were not significantly different—the mean differences in PER, FER and CER were 1.0 % (CI −3.4 to 5.4 %, *p* = 0.858), 0.6 % (−3.8 to 5.0, *p* = 0.943) and 1.7 % (−2.3 to 5.6 %, *p* = 0.576), respectively. It appears that when offered pair-wise choices, cats mixed a diet that was as close as possible to the self-selected diet composition. More remarkable, however, was the finding that the diet macronutrient composition averaged over each of the 3 day cycles of the pair-wise selection phase (Pair Average) is superimposed (yellow dot, Fig. 2) on the compositions selected by naïve and experienced self-selectors with no statistically significant differences between the phases (*p* ≥ 0.248; the largest mean difference was found between ‘Pair Average’ and NSS for CER of 2.2 % (CI −1.0 to 5.6 %)).

### Experiment 2: one dry and three wet foods

**Fig. 3:**
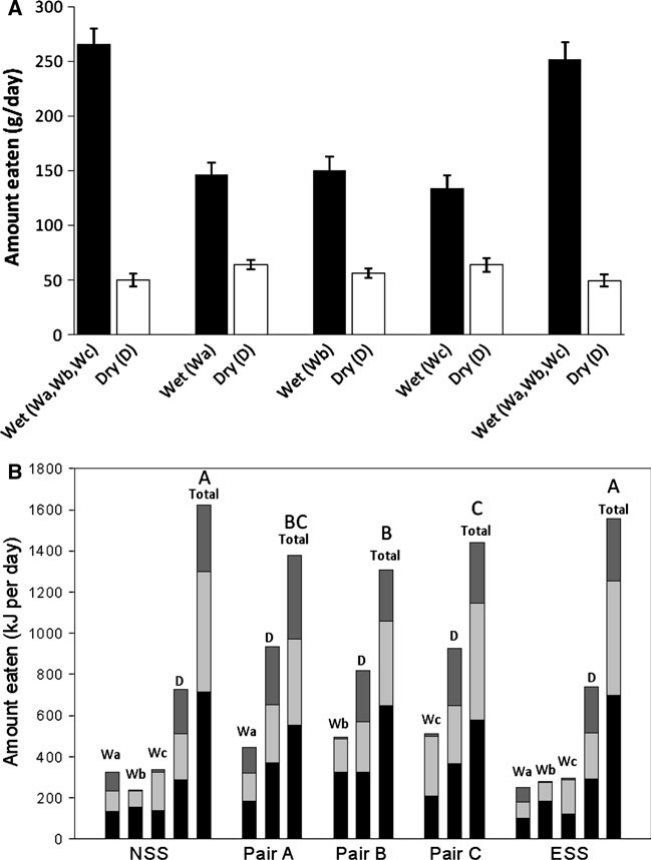
**a** Mean (±95 % confidence interval) food intake and **b** mean amounts of protein (*black*), fat (*light grey*) and carbohydrate (*dark grey*) energy ingested from the wet (Wa, Wb, Wc) and dry (D) foods offered to cats during the naïve self-selection (NSS), pair-wise choices (Pair A, Pair B, Pair C) and experienced self-selection (ESS) phases in experiment 2. Letters *A*–*C* *above the Total bar* (**b**) indicate statistically homogenous groups for total energy intake (i.e. energy intake in phases with the *same letter* are not significantly different)

**Fig. 4:**
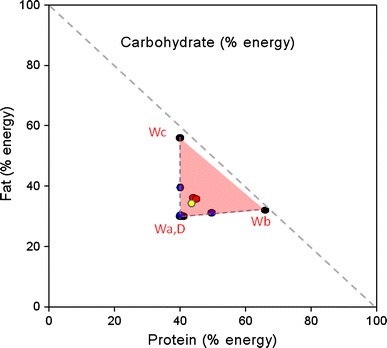
RMT plot for experiment 2 with *black circles* showing the proportional composition of protein, fat and carbohydrate in wet (Wa-Wc) and dry (D) experimental foods. The *pink shaded region* shows the area accessible to self-selecting cats with simultaneous access to the dry and wet foods. *Red circles* show the intake points selected in the naive and experienced self-selection phases and *blue circles* show the macronutrient profile of the diet composed during the pair-wise choices phase (each pair-wise choice is joined by a *dotted blue line*). The diet macronutrient composition averaged over each of the 3-day cycles of the pair-wise selection phase is indicated by the *yellow circle*

Similar to experiment 1, the cats did not appear to be selecting food at random and consuming a similar quantity of food from each of the bowls provided since a greater quantity of wet food than dry food was consumed in each of the phases. In addition, simulation analyses revealed the macronutrient profile obtained in the experiment was significantly different to the profile that would have resulted from random food intake (*p* < 0.001). In this experiment wet food intake was somewhat lower during the pair-wise phase (accounting for ~70 % of total food intake) compared to the NSS and ESS phases (where wet food constituted ~84 % of total food intake) (Fig. 3a). The breakdown of macronutrient energy intake for each of the foods in each phase as well as total energy intake is shown in Fig. 3b. Total macronutrient intake from wet and dry foods amounted to 716 kJ (CI 638–793 kJ) and 703 kJ (630–777 kJ) of protein, 585 kJ (524–647 kJ) and 557 kJ (499–615 kJ) of fat and 281 kJ (215–367 kJ) and 269 kJ (208–350 kJ) of carbohydrate in the NSS and ESS phases, respectively. Figure 4 shows the macronutrient compositions of the diet mixed by naïve and experienced self-selecting cats (red dots) which, as seen in experiment 1, were indistinguishable [the mean differences in PER, FER and CER were 0.9 % (CI −2.7 to 4.4 %, *p* = 0.836), 0.6 % (−3.3 to 4.6, *p* = 0.926) and 0.3 % (−4.2 to 4.7 %, *p* = 0.989), respectively). In addition, when offered pair-wise choices (blue dots), cats appeared to mix a diet that was as close as possible to the self-selected diet composition (note that when offered Wa and D cats were constrained to end up at the same point, since the foods were of the same composition) and the Pair Average diet composition (yellow dot) was also extremely close to that of naïve and experienced self-selecting cats—with 95 % confidence the differences between NSS, Pair Average and ESS were within 4.8 % for PER (*p* ≥ 0.321), 5.7 % for FER (*p* ≥ 0.146) and 7.4 % for CER (*p* ≥ 0.0451). Total energy intakes were significantly lower for each of the pair-wise diet choices compared to the naïve and experienced self-selection phases [differences in energy intake between: NSS and Pair A 261 kJ (CI 133–388 kJ, *p* < 0.001); NSS and Pair B 331 kJ (204–459 kJ, *p* < 0.001); NSS and Pair C 201 kJ (74–329 kJ, *p* < 0.001); ESS and Pair A 193 kJ (65–322 kJ, *p* < 0.001); ESS and Pair B 264 kJ (136 to 393 kJ, *p* < 0.001); ESS and Pair C 134 kJ (5 to 263 kJ, *p* = 0.0366)].

### Experiment 3: three wet and three dry foods

**Fig. 5:**
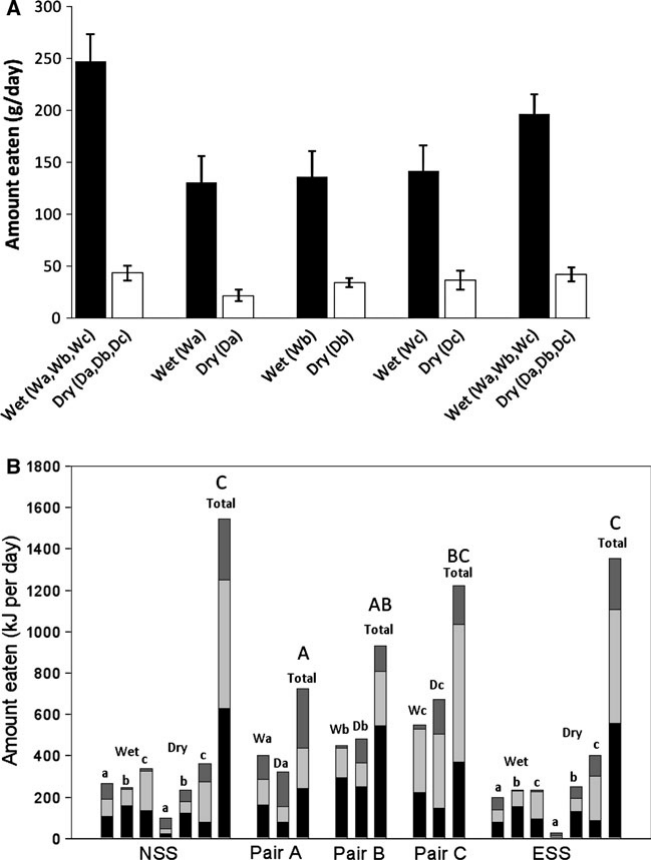
**a** Mean (±95 % confidence interval) food intake and **b** mean amounts of protein (*black*), fat (*light grey*) and carbohydrate (*dark grey*) energy ingested from the wet (Wa, Wb, Wc) and dry (Da, Db, Dc) foods offered to cats during the naïve self-selection (NSS), pair-wise choice (Pair) and experienced self-selection (ESS) phases in experiment 3. Letters *A*–*C above the Total bar* (**b**) indicate statistically homogenous groups for total energy intake (i.e. energy intake in phases with the *same letter* are not significantly different)

**Fig. 6:**
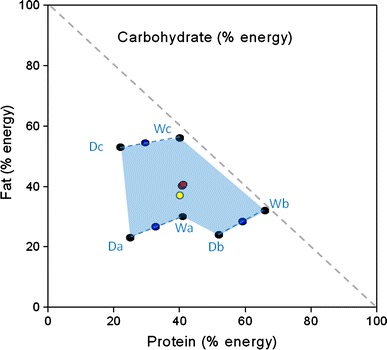
RMT plot for experiment 3 with *black circles* showing the proportional composition of protein, fat and carbohydrate in wet (Wa-Wc) and dry (Da–Dc) experimental foods. The *blue shaded region* shows the area accessible to self-selecting cats with simultaneous access to the dry and wet foods. *Red circles* show the intake points selected in the naive and experienced self-selection phases and *blue circles* show the macronutrient profile of the diet composed during the pair-wise choices phase (each pair-wise choice is joined by a *dotted blue line*). The diet macronutrient composition averaged over each of the 3-day cycles of the pair-wise selection phase is indicated by the *yellow circle*

The quantities of wet and dry food consumed in this experiment were similar to the previous experiments with wet food intake being greater than dry food intake in all phases (Fig. 5a) and different to random intake as determined by simulation analysis (*p* < 0.001). As seen in experiment 2, wet food intake was lower during the pair-wise phase than the NSS and ESS phases although in this experiment wet food intake was maintained at 80–85 % of total food intake. The patterns of macronutrient energy intake for each of the foods in each phase are shown in Fig. 5b and it is notable that cats ate very little of food Da, which was the dry food with very high carbohydrate content (52 % CER), in the naïve and particularly the experienced self-selection phases. Total protein, fat and carbohydrate energy intakes in the NSS and ESS phases were 631 kJ (CI 528–733 kJ) and 560 kJ (461–659 kJ), 621 kJ (475–767 kJ) and 563 kJ (423–704 kJ) and 268 kJ (184–391 kJ) and 223 (151–328 kJ), respectively. Figure 6 shows the macronutrient compositions of the diet mixed by naïve and experienced self-selecting cats (red dots) which, as in experiments 1 and 2, were not statistically different [the mean differences in PER, FER and CER were 0.2 % (CI −11.0 to 11.4 %, *p* = 0.999), 1.0 % (−9.9 to 11.9, *p* = 0.975) and 0.8 % (−10.0 to 11.6 %, *p* = 0.983), respectively). Whilst the food compositions offered in each of the pair-wise food combinations were such that the cats could not mix a diet (blue dots) close to the self-selected composition, the Pair Average macronutrient composition (yellow dot) was not significantly different from that selected in NSS and ESS (largest difference in PER was between Pair Average and NSS of 1.0 %, CI −8.6 to 10.6 %, *p* = 0.967; largest difference in FER was between Pair Average and ESS of 3.8 %, CI −5.6 to 13.1 %, *p* = 0.61; largest difference in CER was between Pair Average and ESS of 4.6 %, CI −4.6 to 13.8 %, *p* = 0.473). Total energy intakes for pair A and pair B were significantly lower than during the NSS and ESS phases (differences in energy intake between: NSS and Pair A 781 kJ, CI 390–1173 kJ, *p* < 0.001; NSS and Pair B 574 kJ, CI 183–965 kJ, *p* < 0.001; ESS and Pair A 612 kJ, CI 220–1003 kJ, *p* < 0.001; ESS and Pair B 404 kJ, CI 13–796 kJ, *p* = 0.039) and energy intake on days Pair A was offered was significantly lower than on days Pair C was offered (500 kJ, CI 84 to 915 kJ, *p* = 0.009) (Fig. 5b).

### Compilation of experiments 1–3

**Fig. 7:**
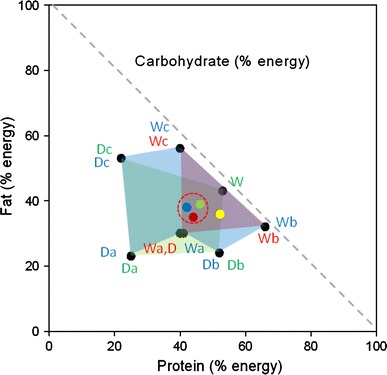
Compilation RMT for experiments 1–3. *Black circles* indicate the diet compositions, with the *colours of the lettering next to each circle* coding the experiment—*green* experiment 1, *red* experiment 2, *blue* experiment 3. The colours used to define the available regions in diet composition space are as in the respective plots in Figs. 2, 4 and 6, with colour blending where regions overlapped. The *green, red and blue circles* within the *dashed red circle* show the overall average macronutrient composition selected across NSS, Pair Average and ESS for experiments 2, 4 and 6, respectively. The *yellow circle* represents the macronutrient composition reported previously for adult domestic cats offered choices of wet foods (Hewson-Hughes et al. 2011)

As can be seen in Table 2, the absolute intake (g day) of each macronutrient was remarkably consistent across the naive and experienced self-selection phases of experiments 1–3. The overall average macronutrient composition selected across NSS, Pair Average and ESS for each experiment was calculated and mixed models analyses performed to test for convergence across experiments. Figure 7 provides a compilation RMT for these three data sets and shows that even though the dietary options were very different in each of the three experiments, the diet compositions achieved were remarkably similar (Expt. 1, 46/39/15; Expt. 2, 44/35/21; Expt. 3, 42/38/20). Furthermore, whilst there were some statistically significant differences between experiments these differences were small. Thus, the PER selected in experiments 1 and 2 was significantly higher than in experiment 3 but at most amounted to a mean difference of only 4.3 % (CI 1.4–7.2 %, *p* = 0.0015) between experiments 1 and 3 whilst the FER was significantly higher in experiments 1 and 3 compared to experiment 2 with the biggest mean difference of 3.7 % (CI 1.3 to 6.1 %, *p* < 0.001) between experiments 1 and 2. Similarly, the CER selected was significantly higher in experiment 2 and 3 than experiment 1 with the largest mean difference of 5.5 % (CI 2.3–8.6 %, *p* < 0.001) between experiments 1 and 2.

### Experiment 4: one wet and one dry food

**Fig. 8:**
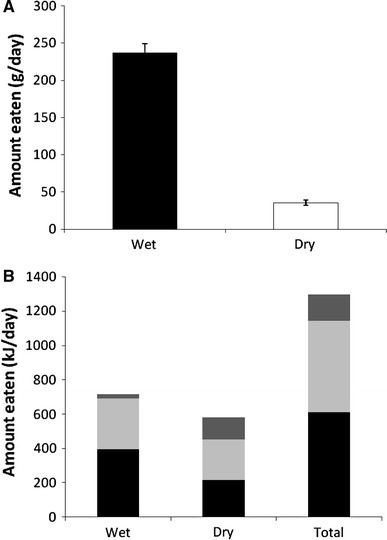
**a** Mean (±95 % confidence interval) food intake and **b** mean amounts of protein (*black*), fat (*light grey*) and carbohydrate (*dark grey*) energy ingested by cats offered one wet (Wd) and one dry (Dd) food in experiment 4

The quantities of wet and dry food consumed in this experiment were similar to experiments 1–3 with wet food accounting for ~87 % of total food intake (Fig. 8a). The macronutrient energy intakes obtained from the wet and dry foods as well as in total are shown in Fig. 8b. The combined intake from the wet and dry foods amounted to 609 kJ (CI 541–678 kJ) of protein, 535 kJ (472–597 kJ) of fat and 154 kJ (120–187 kJ) of carbohydrate giving rise to a diet composition of 48/41/11 (PER/FER/CER; 95 % CI for PER 45.6–49.2 % and for CER 9.6–13.2 %, FER was fixed).

## Discussion

This series of experiments examined the ability of cats to regulate macronutrient intake when provided with foods that not only differed in macronutrient composition, but also in moisture content and consequently in texture and energy density. When the results of experiments 1–3 are superimposed on a single RMT (Fig. 7), where overlapping regions in diet composition space across these three experiments are covered, it can be seen that self-selecting cats in all three experiments achieved remarkably similar diet compositions in terms of the proportions of protein, fat and carbohydrate selected when offered very different combinations of wet and dry foods. Whilst not identical, these profiles accord well with the target composition reported previously (52/36/12) for adult domestic cats offered choices of wet foods (Hewson-Hughes et al. 2011; yellow dot in Fig. 7) and provide further evidence of the cats’ ability to regulate their macronutrient intake, even when provided with foods of very different macronutrient and moisture content simultaneously. Hence, achieving this regulatory outcome involved cats eating different amounts and proportions of foods according to nutrient content, not whether wet or dry. This conclusion is supported by simulations which indicated that had the cats eaten a fixed amount from each bowl of food offered, the macronutrient composition of the resulting diet would have been significantly different to the actual compositions selected and the target macronutrient profile.

Interestingly, the macronutrient profile of the diets composed by domestic cats in the present experiments and previously (Hewson-Hughes et al., 2011) are similar to that reported for free-ranging feral cats (52/46/2; Plantinga et al., 2011), indicating that domestic cats have retained the capacity to regulate macronutrient intake to closely match the ‘natural’ diet of their wild ancestors, even though the manufactured foods provided to domestic cats bear little resemblance to the natural foods (e.g. small vertebrate prey). The macronutrient with the biggest discrepancy between our studies and the reported natural diet of feral cats is carbohydrate. Previously we reported that in achieving their target macronutrient intake cats consumed ~8 g/day carbohydrate (Hewson-Hughes et al. 2011), while in the present experiments cats consumed ~13–20 g/day (Table 2). Eisert (2011) calculated that the maximal amount of digestible carbohydrate (from glycogen and gut contents) a cat could derive from consuming (carbohydrate-loaded) rodent prey is ~2.1 g per day.

The prey-based natural diet of a hypercarnivore such as the cat supplies insufficient carbohydrate to meet the metabolic demands for glucose required, for example, by the brain, and this demand for glucose is met by a high capacity for gluconeogenesis from amino acids (Eisert 2011). Since the cat appears to be metabolically adapted to meet its glucose requirements on a very low carbohydrate diet, it seems unlikely that the higher carbohydrate intakes seen in the present experiments is the result of cats actively seeking higher carbohydrate intake, although this cannot be completely discounted. Thus, having a brain that metabolises glucose like any other animal (Eisert 2011) might have encouraged evolution of broader metabolic use of glucose after generations of access to a higher carbohydrate diet through association with humans. This possibility aside, the cats in the naïve and experienced self-selection phases of these experiments were faced with two or more foods that contained at least 24 % of energy from carbohydrate and intake of only relatively small amounts of these foods would obviously lead to increased carbohydrate intake. Of course, it could be argued that if cats do not ‘need’ dietary carbohydrate then they could have completely avoided these foods, but this does not allow for sampling errors or that animals may have an adaptive strategy of actively sampling available foods to assess their nutritional value or potentially toxic nature (Day et al. 1998). Under either scenario (sampling errors or adaptive sampling), ingesting any of a high-carbohydrate diet will boost intake beyond that possible on a rodent-based natural diet. Although no food type was avoided completely, nonetheless, it is interesting to note that the intake of food Da (the dry food with the highest carbohydrate content, 52 % CER) was very low, particularly in the ESS phase, suggesting that cats had learnt to avoid eating this food. Given the availability of a number of relatively high carbohydrate foods the cats could have consumed much greater amounts of carbohydrate, but in fact ingested ~20 g on average, which is entirely consistent with our previous finding of a 300 kJ/day carbohydrate ceiling (~20 g/day) limiting further food intake (Hewson-Hughes et al. 2011). Furthermore, in situations where cats were offered only two foods (one wet and one dry) that were nutritionally complementary to the previously identified target (i.e. Pair B in experiment 1 and the foods in experiment 4), the cats composed diets that were lower in CER and similar to the target (53/34/13 in experiment 1 and 48/41/11 in experiment 4 compared to target of 52/36/12, Hewson-Hughes et al. 2011).

A particularly notable finding was the convergence upon the same diet composition in the naïve and experienced self-selection phases (where cats were offered all foods simultaneously on the same day) as well as over the course of the 3-day cycles in the pair-wise choice phases of each experiment. We observed this phenomenon previously in sequentially (i.e. 3 foods offered over 3 days—one food/day) and simultaneously (i.e. 3 foods offered on same day) self-selecting cats offered 3 wet foods, but not in experiments where cats were offered 3 dry foods (Hewson-Hughes et al. 2011). This difference between wet and dry foods was explained in terms of cats being unable to avoid the carbohydrate ceiling when forced to switch between dry foods each day over a 3-day period and as a result they under-ate protein relative to simultaneous self-selecting cats (i.e. this was a consequence of the macronutrient composition of the dry foods rather than a property of dry food per se). This phenomenon of regulating to a target macronutrient intake when provided with nutritionally complementary foods simultaneously or at fixed time intervals is not a peculiarity of the cat since it has also been observed in migratory locust (*Locusta migratoria*) nymphs (Chambers et al. 1998). Obviously, it would be interesting to establish if this nutritional regulatory capacity is more widely held across other species.

These studies clearly demonstrate that cats regulate their macronutrient intake even when provided with foods that differ not only in macronutrient composition, but also in their physical characteristics, such as texture and water content. Furthermore, our present results highlight that providing nutritionally complementary wet and dry foods offers cats the opportunity to mix a diet that meets their macronutrient target.
